# Comparative evaluation of protective immunity against *Francisella tularensis* induced by subunit or adenovirus-vectored vaccines

**DOI:** 10.3389/fcimb.2023.1195314

**Published:** 2023-05-25

**Authors:** Mengsu Zhao, Yanfang Zhai, Xiaodong Zai, Yunyun Mao, Enbo Hu, Zhaodong Wei, Yan Li, Kai Li, Yanhong Liu, Junjie Xu, Rui Yu, Wei Chen

**Affiliations:** Institute of Biotechnology, Academy of Military Medical Sciences, Beijing, China

**Keywords:** *Francisella tularensis (F. tularensis)*, subunit vaccine, Ad5-vectored vaccine, immunization, protection

## Abstract

Tularemia is a highly contagious disease caused by infection with *Francisella tularensis* (*Ft*), a pathogenic intracellular gram-negative bacterium that infects a wide range of animals and causes severe disease and death in people, making it a public health concern. Vaccines are the most effective way to prevent tularemia. However, there are no Food and Drug Administration (FDA)-approved *Ft* vaccines thus far due to safety concerns. Herein, three membrane proteins of *Ft*, Tul4, OmpA, and FopA, and a molecular chaperone, DnaK, were identified as potential protective antigens using a multifactor protective antigen platform. Moreover, the recombinant DnaK, FopA, and Tul4 protein vaccines elicited a high level of IgG antibodies but did not protect against challenge. In contrast, protective immunity was elicited by a replication-defective human type 5 adenovirus (Ad5) encoding the Tul4, OmpA, FopA, and DnaK proteins (Ad5-Tul4, Ad5-OmpA, Ad5-FopA, and Ad5-DnaK) after a single immunization, and all Ad5-based vaccines stimulated a Th1-biased immune response. Moreover, intramuscular and intranasal vaccination with Ad5-Tul4 using the prime-boost strategy effectively eliminated *Ft* lung, spleen and liver colonization and provided nearly 80% protection against intranasal challenge with the *Ft* live vaccine strain (LVS). Only intramuscular, not intranasal vaccination, with Ad5-Tul4 protected mice from intraperitoneal challenge. This study provides a comprehensive comparison of protective immunity against *Ft* provided by subunit or adenovirus-vectored vaccines and suggests that mucosal vaccination with Ad5-Tul4 may yield desirable protective efficacy against mucosal infection, while intramuscular vaccination offers greater overall protection against intraperitoneal tularemia.

## Introduction

*Francisella tularensis* (*Ft*), a nonmotile, intracellular gram-negative coccobacillus, is the causative agent of fatal tularemia disease, which is endemic throughout most of Europe, northern and central Asia and North America (Telford and Goethert, 2020). Natural infection occurs in wild animals, including lagomorphs, rodents, carnivores, ungulates, marsupials, amphibians, birds, fish and invertebrates (Putzova et al., 2016). Transmission to humans also occurs via the bite of disease-carrying arthropods or inhalation and ingestion of infective materials. The main symptoms of tularemia include ulceration at the portal of infectious entry, lymphadenopathy, pneumonia and sore throat, high fevers, or chills, which are closely associated with the infection routes in hosts (Telford and Goethert, 2020). Tularemia pneumonia is less common but more lethal, with a 30–60% mortality rate (Feldman et al., 2001; Sjostedt, 2007).

Among the four accepted subspecies, *Ft* subsp. *tularensis* (type A, including the Schu S4 strain), *Ft* subsp. *holarctica* (type B), *Ft* subsp. *mediasiatica* and *Ft* subsp. *Novicida (* Larson et al., 2014), the live vaccine strain (LVS) derived from type B *Ft* is attenuated in humans but highly virulent in mice, causing a disease that closely resembles human tularemia (Elkins et al., 2003). The attenuation of the multideletional mutant *Ft* LVS strain remains undefined (Pechous et al., 2009); the strain exhibits notable toxicity (Saslaw et al., 1961) and provides poor protection against high-dose aerosol challenge (McCrumb, 1961); and the strain is used as a vaccine against tularemia only under special circumstances in the United States (Jia et al., 2013). *Ft* LVS is however extensively used to establish a helpful experimental infection model in mice to test potential vaccine candidates and vaccination strategies against *Ft (* Ashtekar et al., 2012), and the strain can be handled at the biosafety level 2 (BLS-2) level. In addition, no licensed or FDA-approved vaccines are currently available. Thus, developing a safe and effective vaccine against *Ft* is emerging as a focus to confront this potential biosafety threat.

*Francisella* vaccine development has been ongoing since the 1920s, including the production of heat-, chemical- (Foshay et al., 1942; Foshay, 1950), or ionizing radiation- (Gordon et al., 1964) killed whole-cell vaccines, live attenuated vaccines (Oyston and Quarry, 2005; Hepburn et al., 2006; Bakshi et al., 2008; Barry et al., 2009; Mahawar et al., 2013; Marohn and Barry, 2013; Chu et al., 2014), subunit vaccines and recombinant vector-based vaccines (Mansour et al., 2018). Moreover, a number of *Ft* antigens have the potential for use in tularemia vaccines, including carbohydrates, lipopolysaccharide (LPS) (Fulop et al., 1995; Fulop et al., 2001; Conlan et al., 2002), outer membrane proteins (OMPs, such as OmpA, FopA (Hickey et al., 2011; Post et al., 2017) and Tul4 (Huntley et al., 2007; Kaur et al., 2012)) and intracellular heat shock proteins (HSPs, such as DnaK). However, no vaccines are licensed for human use, and the limited protection offered by the current vaccines indicates the need for the development of improved *Ft* vaccines with higher levels of safety and protection.

In the present study, we performed a comparative evaluation of protective immunity against tularemia provided by subunit or Ad5-vectored vaccines, and suggested that mucosal vaccination using Ad5-Tul4 may provide desirable protective efficacy against the most severe *Ft*-induced disease, respiratory tularemia.

## Results

### Identification of potential antigens for tularemia vaccine development

**Figure 1:**
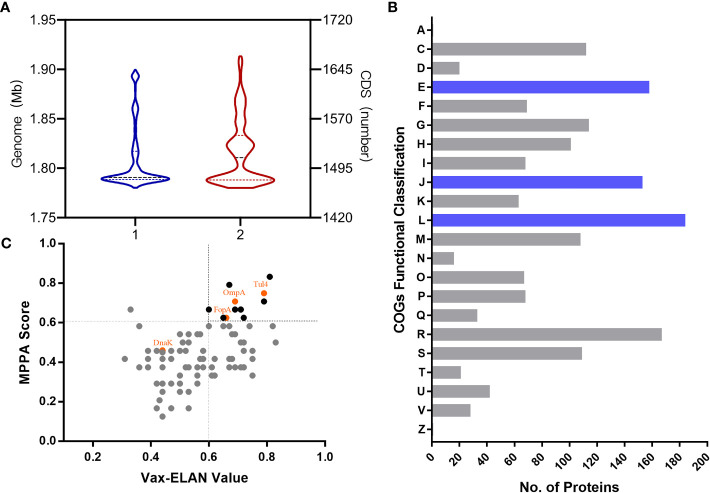
Distribution of the genome and functional proteins in *Ft.* **(A)** The distribution of representative strains of *Ft* according to genome size and numbers of CDS. **(B)** Functional classification of proteins identified according to Clusters of Orthologous Groups (COGs). A, RNA processing and modification; C, Energy production and conversion; D, Cell cycle control, cell division, chromosome partitioning; E, Amino acid transport and metabolism; F, Nucleotide transport and metabolism; G, Carbohydrate transport and metabolism; H, Coenzyme transport and metabolism; I, Lipid transport and metabolism; J, Translation, ribosomal structure and biogenesis; K, Transcription; L, Replication, recombination and repair; M, Cell wall/membrane/envelope biogenesis; N, Cell motility; O, Posttranslational modification, protein turnover, chaperones; P, Inorganic ion transport and metabolism; Q, Secondary metabolite biosynthesis, transport and catabolism; R, General function prediction only; S, Function unknown; T, Signal transduction mechanisms; U, Intracellular trafficking, secretion, and vesicular transport; V, Defense mechanisms; Z, Cytoskeleton. **(C)** Distribution of the main “probable antigens” according to MPPA score and Vax-ELAN value.

First, immunoinformatic analysis was implemented for systematic identification of potential epitopes and antigens for tularemia vaccine development. The genome information of 756 strains of *Francisella* with a normal genome size and number of protein-encoding sequences (CDS) was obtained from the NCBI database. The mean genome size was 1.80 Mb (1.78-1.9 Mb), and the average number of CDS was 1,519 (**Figure 1A**). Then, the Clusters of Orthologous Groups (COG) functional annotation of 2,188 coding sequences of the representative highly virulent *Ft* subsp. *tularensis* Schu S4 strain was applied, and 1,703 identified proteins had known functional classifications, covering 22 functional classifications in COG. Among them, the clusters for “translation, ribosomal structure and biogenesis” (184, 10.8%), “replication, recombination and repair” (158, 9.2%) and “amino acid transport and metabolism” (153, 9.0%) were the top three largest functional groups (**Figure 1B**). Then, the 95 proteins that carried one or more protective signatures recurring in known bacterial protective antigens (Altindis et al., 2015) were further analyzed using two reverse vaccinology tools, including the self-developed Multifactor Prediction of Protective Antigens (MPPA) platform based on subcellular localization, antigen similarity, antigenicity, mature epitope density, virulence, and adhesion probability (Zai et al., 2021) and the Vax-ELAN pipeline based on subcellular localization, transmembrane helix prediction, adhesion property, non-homology with host proteins, etc (Rawal et al., 2021) (**Figure 1C**). The potential immunogenic antigens—Tul4, OmpA, FopA, and DnaK—had both a high MPPA score and Vax-ELAN value, indicating likely involvement in antigenicity and virulence, and were further assessed as vaccine candidates.

### Tul4, FopA, and DnaK subunit vaccines conferred immunogenic potential but little protection against challenge

**Figure 2:**
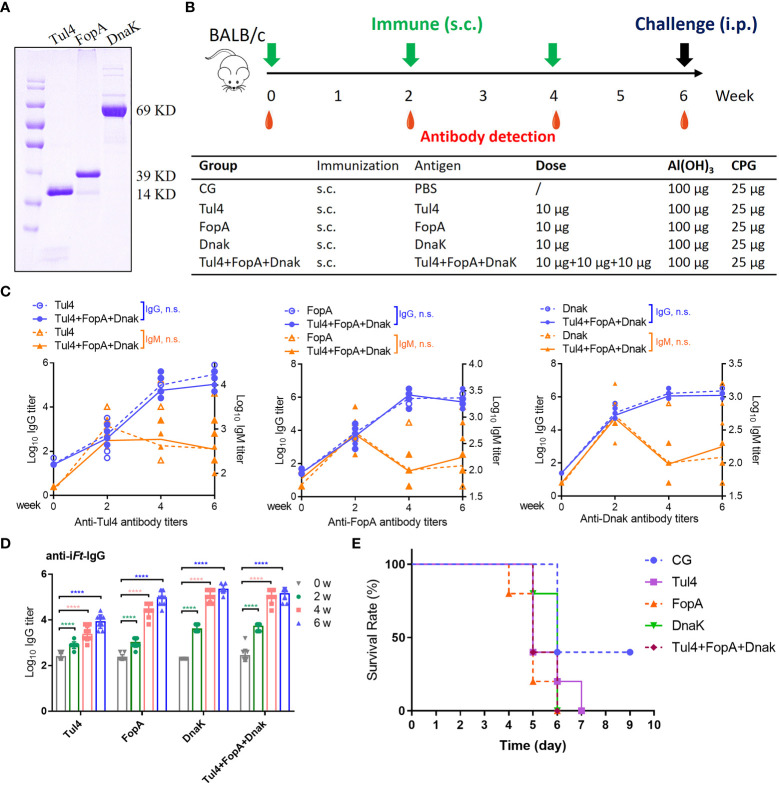
Immune reactivity to Tul4, FopA, and DnaK subunit vaccines. **(A)** The recombinant codon-optimized Tul4, FopA, and DnaK were purified and examined by Coomassie blue staining. **(B)** Mice were immunized via the s.c. route with Tul4, FopA, DnaK or Tul4+FopA+DnaK (cocktail) on days 0, 14 and 28. Serum samples were collected at the indicated time. **(C)** Induction of equivalent Tul4-, FopA-, and DnaK-specific serum IgG and IgM antibody responses following s.c. immunization with cocktail subunit vaccines. Comparisons between the two groups were analyzed by unpaired t tested. **(D)** The levels of anti-i*Ft* IgG in the serum were determined by ELISA. **(E)**The survival rate after challenge with 2×10^3^ CFU of *Ft* LVS via the i.p. route. CG, control group; n.s., not significant, P > 0.05; ****P < 0.0001.

Codon-optimized Tul4, FopA, and DnaK were purified and examined for immunogenicity when formulated as subunit vaccines (**Figure 2A**). Mice that were subcutaneously (s.c.) immunized three times 2 weeks apart with 10 μg of recombinant Tul4, FopA, or DnaK together with Al(OH)_3_+CPG adjuvants had high self-matched IgG antibody responses, while cocktail (Tul4+FopA+DnaK) immunization showed no superiority (**Figures 2B, C**). The IgG levels in vaccinated mice rose steadily, while the IgM levels peaked 2 weeks after the first immunization and subsequently declined in all experimental groups (**Figure 2C**). Moreover, indistinguishable increased anti-i*Ft* IgG antibody responses were observed with increasing time among the different groups (**Figure 2D**). However, all immunized mice succumbed to infection by 6-7 days after challenge, almost without exception (**Figure 2E**). In agreement with previous studies (Kaur et al., 2012), these results demonstrated that recombinant Tul4, FopA, and DnaK subunit vaccines conferred immunogenic potential but little protection against challenge with high-activity *Ft* LVS.

### Effectiveness of a single dose of Ad5-based vaccines in inducing a protective response against *Ft* LVS infection

**Figure 3:**
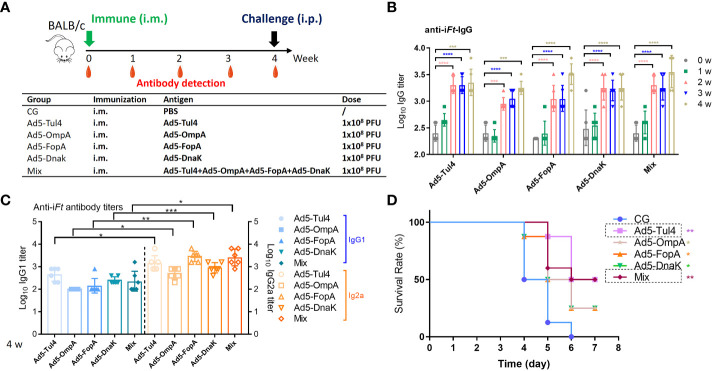
Immune reactivity to recombinant Ad5-Tul4, Ad5-OmpA, Ad5-FopA, and Ad5-DnaK vaccines. **(A)** Mice were immunized i.m. on day 0 with Ad5-Tul4, Ad5-OmpA, Ad5-FopA, or Ad5-DnaK; serum was then collected at the indicated time for antibody detection. **(B, C)** Anti-i*Ft* IgG **(B)** and IgG1 and IgG2a **(C)** were analyzed by ELISA. Comparisons between IgG1 and IgG2a were analyzed by unpaired t tested. **(D)** The survival rate after challenge with 2×10^3^ CFU of *Ft* LVS via the i.p. route. CG, control group. The significance of differences was calculated by comparison with the control group. *P < 0.05; **P < 0.01; ***P < 0.001; ****P < 0.0001.

Then, the recombinant Ad5-Tul4, Ad5-OmpA, Ad5-FopA, and Ad5-DnaK vaccines were further analyzed for immune reactivity. Mice receiving a single dose of Ad5-Tul4, Ad5-OmpA, Ad5-FopA, or Ad5-DnaK by the i.m. route generated increased anti-i*Ft* IgG levels with increasing time (**Figures 3A, B**). Coadministration of a mixture of various Ad5-based vaccines also resulted in no significant augmentation of the serum IgG immune response (**Figure 3B**). Furthermore, Ad5-based vaccines generated a Th1-predominated humoral immune response, indicated by higher levels of *Ft*-specific IgG2a antibodies (**Figure 3C**). In addition, splenocytes from mice immunized with Ad5-Tul4 (14 days post immunization) responded to *in vitro* stimulation with purified Tul4 fragment 17 (QGSVRLQWQAPEGSK) and to a greater extent fragment 18 (RLQWQAPEGSKCHDT) by producing IFN-γ, demonstrating that the Tul4 antigen was indeed processed and presented to T cells, eliciting a potent cellular immune response (**Supplementary Figures S1A, B**). Remarkably, 50% of the mice after single immunization with Ad5-Tul4 or Ad5-mixture were protected against *Ft* LVS challenge, while a relatively lower survival rate (25%) was observed when mice were immunized with Ad5-OmpA, Ad5-FopA, or Ad5-DnaK (**Figure 3D**). Taken together, these results demonstrated the ability of Ad5-based vaccines, especially Ad5-Tul4, to provide efficient protection against *Ft* LVS.

### Intranasal immunization with Ad5-Tul4 using a prime-boost strategy conferred protection against infection and reduced the bacterial dissemination in mouse tissues

**Figure 4:**
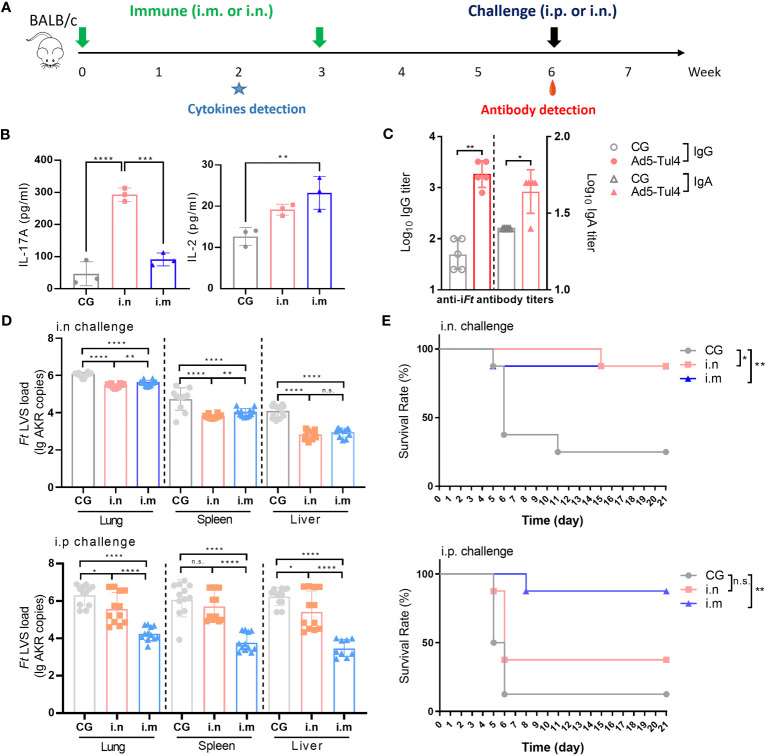
Immune reactivity to Ad5-Tul4 confers protection against *Ft* LVS challenge. **(A)** Mice were immunized intranasally or intramuscularly on days 0 and 21 with Ad5-Tul4. The mice in the control group (CG) received PBS. Three weeks after the last immunization, mice were challenged with 2×10^3^ CFU of *Ft* LVS via either the i.n. route or i.p. route. **(B)** Induction of cytokines after immunization via the i.n. route or i.m. route. Splenocytes from mice immunized via the i.n. route or i.m. route with Ad5-Tul4 (14 days post-immunization) were extracted and then stimulated with Tul4, and then the indicated cytokines were quantified by flow cytometry. **(C)** Anti-i*Ft* IgG and IgA in the bronchoalveolar lavage fluid of intranasally immunized mice were quantified by ELISA. Comparisons between the two groups were analyzed by unpaired t tested. **(D)** Immunization resulted in a reduced bacterial dissemination. The relative levels of *Ft* LVS-specific AKR DNA in the spleen, liver and lung of mice infected via either the i.n. route (top) or i.p. route (bottom) (n = 12). **(E)** Immunization conferred protection against challenge. Mice were challenged with *Ft* LVS via either the i.n. route (top) or i.p. route (bottom), and survival was monitored for 21 days (n = 10). n.s., not significant, P > 0.05; *P < 0.05; **P < 0.01; ***P < 0.001; ****P < 0.0001.

To determine the optimal immunization regimen for protection, groups of mice were immunized via the i.n. route and i.m. route with Ad5-Tul4 on days 0 and 21 (**Figure 4A**). Two intranasal immunizations induced IL-17A production, while intramuscular immunizations tended to elicit a higher level of IL-2 production, indicating that Th17- and Th1-predominated humoral immune responses were induced by i.n. and i.m. immunization, respectively (**Figure 4B**). Mice immunized with Ad5-Tul4 via the i.n. route produced higher levels of anti-i*Ft* IgG and IgA in bronchoalveolar lavage fluid, highlighting that intranasal delivery induced a remarkable localized mucosal immune response (**Figure 4C**). Immunized and nonimmunized mice were then challenged via the respiratory route or intraperitoneal route to mimic mucosal and systemic infection, respectively, with *Ft* LVS (2×10^3^ CFU) 3 weeks after the second immunization. Five days post-infection, the spleen, liver and lung were harvested, and the expression of *Ft* LVS-specific AKR DNA was assessed using real-time PCR as a measurement of bacterial burden in the tissues. Compared to the i.m. immunized mice, i.n.-immunized mice showed a significantly lower bacterial dissemination in the lung and spleen upon i.n. challenge, with a 73.86% *Ft* LVS load reduction in the lung, 92.81% in the spleen and 95.16% in the liver (**Figure 4D**, **Supplementary Table S1**), demonstrating improved protection with respiratory vaccination against intranasal challenge. However, the effect on of i.n. immunization on bacterial clearance was poor upon i.p. challenge, the *Ft* LVS load reduction rates were 52.48%, 45%, and 17.38% in the lung, spleen, and liver, respectively, much lower than that with i.m. immunization (*Ft* LVS load reduction rates were 99.31%, 99.70%, and 99.81%, respectively) (**Figure 4D**, **Supplementary Table S1**). Consistently, approximately 80% protection against i.n. challenge was provided by both i.n. and i.m. immunization, while only i.m.-immunized mice survived i.p. infection (**Figure 4E**). Taken together, these results demonstrated that a regimen consisting of two intranasal immunizations with Ad5-Tul4 was more effective in protecting mice from respiratory infection, while intramuscular immunizations were superior for resisting systemic *Ft* colonization.

## Discussion

*Ft* has been characterized with extreme virulence and ease of aerosol transmission (Elkins et al., 2003). In the present study, antigen-specific systemic antibody production and cell-mediated immune responses induced by two forms of vaccines expressing distinct immunodominant antigens of *Ft* were analyzed. Ad5-based vaccines exhibited an advantage in terms of effective protection against challenge. Specifically, a single dose of Ad5-Tul4 protected 50% of mice, while two doses protected 80% of mice from *Ft* challenge. Moreover, mucosal Ad5-Tul4 prime-boost administration via the i.n. route induced strong immune responses and conferred protective immunity against *Ft* LVS intranasal challenge, while intraperitoneal inoculation provided broader protection against both respiratory and systemic infection. Our results supported the potential of Ad5-Tul4 as a nasal vaccine to combat respiratory *Francisella* infection.

Immunization with Tul4, FopA or DnaK alone induced robust antibody responses, yet their combination afforded no greater production, similar to the case for Ad5-based vaccines, probably owing to the similar immune responses elicited by these antigens. Thus, the generation of protective immunity against *Ft* likely requires the recognition of multiple antigenic determinants capable of stimulating distinct humoral and cell-mediated immune responses.

Several studies have shown that subunit vaccines have a limited protective effect. For example, Ashtekar et al. showed that recombinant DnaK or Tul4 together with the adjuvant GPI-0100 protected only 35% of mice from highly lethal challenge (8×10^6^ CFU) (Ashtekar et al., 2012). Humanized mice immunized with a cocktail of recombinant Tul4 and FopA showed only a prolonged median survival time but no statistically significant difference in the survival rate between the immunized and control groups (Oh et al., 2018). The failure to identify protective subunit vaccines might reflect the requirement for CD8^+^ T-cell responses (Griffin et al., 2007). Further efforts have been made to use different antigen delivery platforms, such as tobacco mosaic virus (TMV)-based vaccines and adenovirus-based vaccines (Kaur et al., 2012). The potential reason for this differential protective efficacy may be the virulence discrepancy of the bacteria used based upon growth conditions, e.g., laboratory media and growth stages, as well as the host sensitivity. In particular, C57BL/6 mice are known to be more susceptible than BALB/c mice to *Ft* infection (Oh et al., 2018).

One of the most likely routes of *Ft* infection is the intranasal route, which results in pneumonic tularemia with a mortality rate of up to 60% without therapeutic intervention. Bacteria dissociate from the respiratory tract, and continued replication in other organs results in septicemia and cytokine storms, which lead to overwhelming tissue damage and eventual death (Nicol et al., 2021). Our results demonstrated important differences between routes of vaccination, which has been appreciated in multiple models of infectious diseases that lack successful vaccines today, including chlamydia, AIDS, and tuberculosis (Hu et al., 2013; Beverley et al., 2014; Lai et al., 2015; Nicol et al., 2021). Moreover, it is now well established that within the lung microenvironment, pathogens and their hosts interact in complex ways not observed in other organs. Thus, vaccines administered through the respiratory tract have advantages in enhancing the first line of defense against mucosal infection with *Ft.*

Generating a vaccine without safety concerns for adverse effects is a focus for *Ft* vaccine development. Ad5 vectors have been widely used for vaccine development, despite setbacks in Merck Ad5/HIV trivalent vaccine which has shown no protection but outright harm against HIV infection (Cohen, 2007; Sekaly, 2008), and prospective studies demonstrating the provided high levels of protection against various pathogens, such as Ebola virus (Wu et al., 2016) and Zika virus (Guo et al., 2018), revealed the feasibility of using Ad5 vectors for antigen delivery. Of note, aerosolized Ad5-nCoV has been shown to have good safety and immunogenicity profiles in clinical trials (Wu et al., 2021), highlighting that vaccines based on the Ad5 delivery platform have great potential for respiratory administration.

There are limitations in this study that need to be improved. For example, the conclusions would be more precise if CG mice received empty Ad5 rather than PBS as a negative control.

In summary, our study identified an intranasal Ad5-Tul4 vaccine that elicited desirable protection against respiratory LVS infection, which is worth further investigation in human clinical trials.

## Materials and methods

### Bacterial strains

The *Francisella tularensis* LVS strain was obtained from the Institute of Microbiology and Epidemiology, Beijing, China (Duan et al., 2000), handled in a BSL-2 laboratory; cultured on solid medium containing 4% tryptic soy agar (TSA, Solarbio, T8650), 0.2% L-cysteine hydrochloride monohydrate (Solarbio, C0011), and 7% defibrinated rabbit blood (Shanghai Yuanye Bio-Technology Co Ltd, MP20025); and grown for 12-15 h at 37°C in an atmosphere of 5% CO_2_. Then, a bacterial suspension was made in sterile PBS, followed by OD_600_ measurement (one OD_600_ corresponded to 3× 10^8^ CFU/ml). The bacteria were diluted to achieve a final concentration of 2× 10^4^ CFU/ml or 4× 10^4^ CFU/ml, and these suspensions were used in all challenge experiments.

### Production of recombinant Tul4, FopA, and DnaK proteins

pTIG-Trx vectors were constructed with the codon-optimized Tul4, FopA, and DnaK genes. Then, the vectors were transferred to *E. coli* BL21 competent cells, and expression was induced by the addition of IPTG (isopropyl-β-d-thiogalactoside) when the OD_600_ reached 0.8-1.0. The identity of the purified rTul4, rFopA, and rDnaK proteins was examined by sodium dodecyl sulfate−polyacrylamide gel electrophoresis (SDS−PAGE) with Coomassie Blue staining. The concentration of each recombinant protein was estimated by a BCA Protein Assay Kit (Thermo Fisher, USA) according to the manufacturer’s instructions.

### Construction of Ad5-based vaccines

Recombinant replication-defective human type 5 adenoviruses were constructed as described previously (Wu et al., 2020). Briefly, codon-optimized genes encoding Tul4, OmpA, FopA, and DnaK were synthesized and cloned into the shuttle plasmid pDC316 with the AdMax adenovirus system (Microbix Biosystem, Canada). HEK293 cells were then cotransfected with the constructed shuttle plasmids together with the backbone plasmid (pBHGloxΔE1, 3Cre). The cells exhibiting obvious cytopathic effects were lysed, and the obtained recombinant adenoviruses were amplified by serial passage in HEK293 cells. The viruses were purified by an Adeno-X™ Virus Purification Kit (BD Biosciences, Clontech) and titrated by an endpoint dilution assay.

### Animal experiments

Animal experiments were performed according to the guidelines of the Institutional Experimental Animal Welfare and Ethics Committee. Female BALB/c mice aged 8-10 weeks (purchased from Vital River Laboratories, Beijing, China) were used for all experimental groups. For subunit vaccine administration, mice were immunized via the s.c. route on days 0, 14 and 28 with 10 μg of the indicated proteins together with 100 μg of Al(OH)_3_ (Alhydrogel^®^2%, Brenntag Biosector, Frederikssund, Denmark) and 25 μg of CpG1826 (5’- TCCATGACGTTCCTGACGTT-3’, Takara Clontech) adjuvants. For Ad5-based vaccine administration, mice were immunized i.m. (100 μl) or i.n. (50 μl, 25 μl per nostril) on day 0 or on days 0 and 21 with 1x10^8^ plaque-forming units (PFU) of the indicated adenoviruses. For challenges, intraperitoneal (i.p.) injection (100 μl, 2×10^4^ CFU/ml) or intranasal inhalation (50 μl, 25 μl per nostril, 4×10^4^ CFU/ml) of *Ft* LVS were applied at the indicated time.

### Quantification of *F. tularensis* burden

*Ft* burden was evaluated with real-time PCR as previously described (Shi et al., 2009). Briefly, total nucleic acids were purified from the lung, spleen or liver harvested from euthanized mice by phenol−chloroform extraction and then quantified by a NanoDrop spectrophotometer (NanoDrop Technologies, Wilmington, DE), followed by dilution to 5 ng/μl. The *Ft*-specific AKR gene was quantified by using a TaqMan Universal Master Mix II Kit (Thermo Fisher, USA). Five microliters of purified DNA was amplified in a 20 μl reaction containing 10 μl of 2×TaqMan Universal Master Mix II, 0.8 μM forward primer, 0.8 μM reverse primer, and 200 nM probe. A forward primer (5’-GCAGGGCGAGCACCATT-3′), reverse primer (5′-ATCTTGCATGGTCACCACTTGA-3’), and probe (5’-FAM-CGATATTTGCCTGTTAGCACTCCT-Tamra-3’) were used. Reactions were incubated at 50°C for 2 min, followed by 95°C for 10 min, and then thermal cycled for 40 cycles (95°C for 15 s and 60°C for 1 min).

### Measurement of antibody levels by ELISA

For measurement of IgG (IgG1 and IgG2a) and IgM titers, serum from immunized mice was serially diluted and added to 96-well microplates (Corning, USA), which were precoated with 2 μg/ml indicated antigen proteins or 4% paraformaldehyde-inactivated *Ft* (i*Ft*) at 4°C overnight. After successive antibody incubation with 1:20000-diluted HRP-conjugated goat anti-mouse IgG, IgG1, or IgG2a (Abcam, UK) and washing with PBST, the assay was then developed for 6 min with 100 μl of TMB substrate solution (Solarbio, China), stopped by the addition of 50 μl of stop solution (Solarbio, China), and measured at 450 nm/630 nm (SPECTRA MAX 190, Molecular Device, USA). The endpoint titer was defined as the highest reciprocal serum dilution that yielded an absorbance > 2-fold over the optical absorbance value of the negative control.

### Cytokines detection

A cytometric bead array (CBA) mouse Th1/Th2/Th17 cytokine kit (BD Biosciences) was used for cytokines detection according to the manufacturer’s protocols. Briefly, a total of 2×10^5^ splenocytes were seeded in 96-well plates and treated with purified Tul4 protein for 72 h at 37°C with 5% CO_2_. Then the supernatant was collected and mixed with Capture Beads and the indicated cytokines were then quantified by flow cytometry.

### T-cell epitope identification

A mouse IFN-γ enzyme-linked immunosorbent spot (ELISpot) kit (MabTech, Sweden) was used for T-cell epitope identification according to the manufacturer’s protocols. Briefly, a total of 2×10^5^ splenocytes from immunized mice were stimulated with 31 synthesized peptides (GL Biochem, Shanghai, 10 μg/ml) and seeded in precoated ELISpot plates for 48 h at 37°C with 5% CO_2_. After successive antibody incubation and washing, the plates were measured on the AT-Spot 2100 reader (Beijing Antai Yongxin Medical Technology, China).

### Statistical analysis

All of the statistical analyses were performed using GraphPad Prism 8.0.2 software. Comparisons among the groups were analyzed by one-way analysis of variance (ANOVA) unless otherwise specified. For survival analysis, the log-rank (Mantel−Cox) test was used. Antibody titer data were log transformed before analysis. Data are shown as the geometric mean with geometric SD. n.s., not significant, P > 0.05; *P < 0.05; **P < 0.01; ***P < 0.001; ****P < 0.0001.

## Data availability statement

The raw data supporting the conclusions of this article will be made available by the authors, without undue reservation.

## Ethics statement

The animal study was reviewed and approved by the Animal Care and Use Committee of the Beijing Institute of Biotechnology.

## Author contributions

JX, RY and WC designed, directed and supervised the entire study. MZ performed most of the experiments and processed data. YZ analyzed the results and wrote the manuscript. XZ performed the immunoinformatic analysis. YM, EH, ZW, YLi, KL, and YLiu assisted in the immunization and evaluation experiments. All authors contributed to the article and approved the submitted version.
