# Supplementary material for: Comparative evaluation of protective immunity against Francisella tularensis induced by subunit or adenovirus-vectored vaccines
Source: Front Cell Infect Microbiol. 2023 May 25;13:1195314. doi: 10.3389/fcimb.2023.1195314 (PMC10248143; doi:10.3389/fcimb.2023.1195314)
Supplement: Supplementary file 1 [file DataSheet_1.docx]

Supplementary Materials for

**Comparative evaluation of protective immunity against *Francisella tularensis* induced by subunit or adenovirus-vectored vaccines**

Mengsu Zhao*^1^, Yanfang Zhai*^1^, Xiaodong Zai^1^, Yunyun Mao^1^, Enbo Hu^1^, Zhaodong Wei^1^, Yan Li^1^, Kai Li^1^, Yanhong Liu^1^, Junjie Xu^1^, Rui Yu^#1^, Wei Chen^#1^

^1^Beijing Institute of Biotechnology, Beijing 10071, China.

*These authors contributed equally to this study

# Address correspondence to：

Prof. Rui Yu, yurui1102@139.com

Prof. Wei Chen, cw0226@foxmail.com

**This file includes:** Supplementary Figure S1 and Supplementary Table S1.

Supplementary Figure S1

**
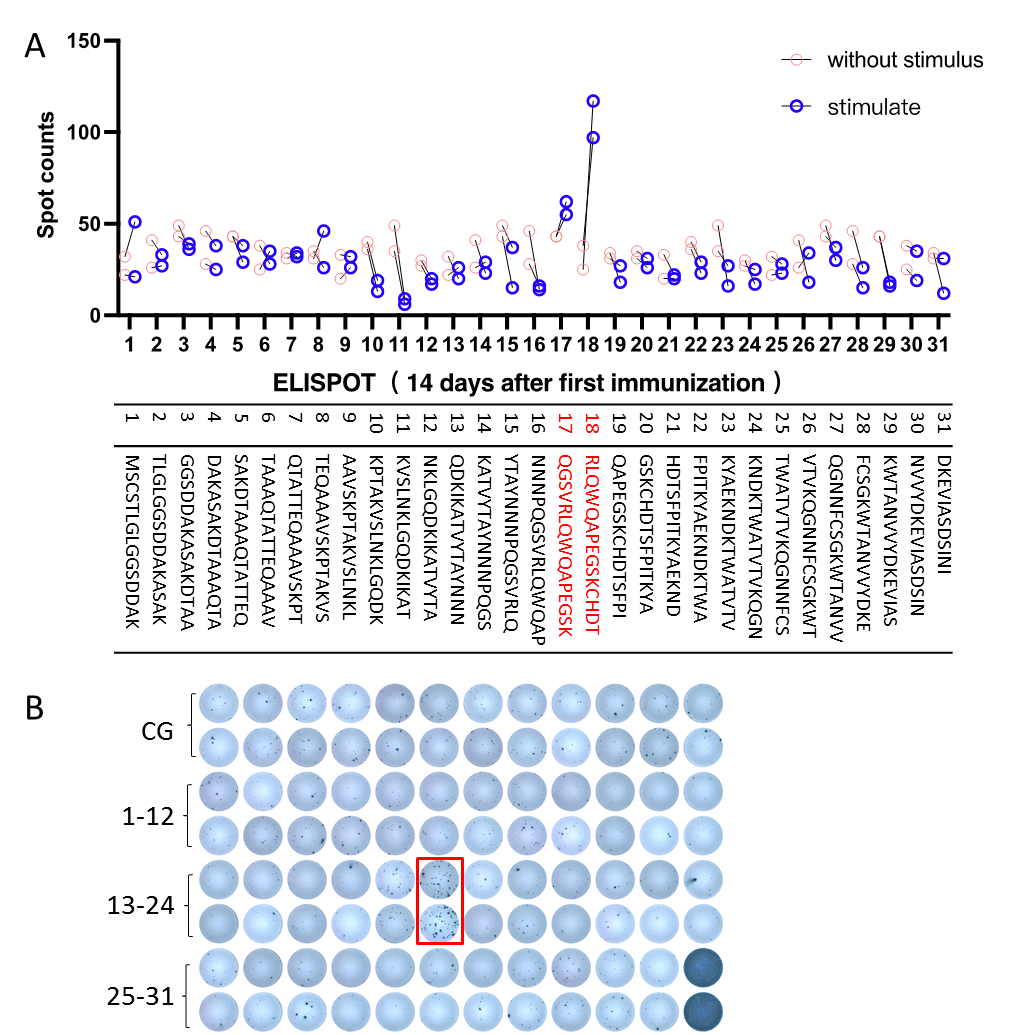
**

**Supplementary Figure S1 Tul4 fragment 17 and fragment 18 were recognized by splenocytes after Ad5-Tul4 immunization.** Spleen cells of Ad5-Tul4-immunized mice were cultured for 18–24 h with a series of Tul4 fragments. Cells in control group wells were cultured with medium alone. The induced IFN-γ levels were measured by ELISpot.

**Supplementary Table S1**

***F. tularensis* LVS load reduction after immunization with Ad5-Tul4 through i.n. or i.m. route.**

|  | |  | | Lung | | | | | | Spleen | | | | | | Liver | | | | | |
| --- | --- | --- | --- | --- | --- | --- | --- | --- | --- | --- | --- | --- | --- | --- | --- | --- | --- | --- | --- | --- | --- |
|  | |  | | CG | | i.n | | i.m | | CG | | i.n | | i.m | | CG | | i.n | | i.m | |
| i.n | | mean | | 1129238.06 | | 295141.425 | | 419893.305 | | 90513.4391 | | 6501.04517 | | 11552.2552 | | 15130.8666 | | 732.3672 | | 973.334818 | |
|  |  | percentage of *Ft* LVS load reduction (/CG) | | / | | 73.86% | | 62.81% | | / | | 92.81% | | 87.23% | | / | | 95.16% | | 93.57% | |
| i.p | | mean | | 3630618.11 | | 1725160.96 | | 24879.4426 | | 3691442.44 | | 2030220.86 | | 11018.2214 | | 2539880.78 | | 2098555.67 | | 4794.65233 | |
|  |  | percentage of *Ft* LVS load reduction (/CG) | | / | | 52.48% | | 99.31% | | / | | 45% | | 99.70% | | / | | 17.38% | | 99.81% | |
